# Comparing Efficacy of Online and In-Person Versions of a Training on U.S. Federal Wage and Hour, Child Labor Laws, and Hazardous Occupations Orders for Secondary School Professionals

**DOI:** 10.3389/fpubh.2016.00075

**Published:** 2016-04-25

**Authors:** Derek G. Shendell, Alexsandra A. Apostolico, Lindsey J. Milich, Alexa A. Patti, Sarah W. Kelly

**Affiliations:** ^1^NJ Safe Schools Program, Center for School and Community-Based Research and Education, Rutgers School of Public Health (SPH), New Brunswick, NJ, USA; ^2^Department of Environmental and Occupational Health, Rutgers School of Public Health (SPH), Piscataway, NJ, USA; ^3^Exposure Measurement and Assessment Division, Environmental and Occupational Health Sciences Institute, Rutgers Robert Wood Johnson Medical School, Piscataway, NJ, USA; ^4^Department of Epidemiology, Rutgers School of Public Health (SPH), Piscataway, NJ, USA

**Keywords:** child labor laws, occupational health and safety, online training, wage and hour laws, young workers

## Abstract

**Background:**

The New Jersey Safe Schools Program (NJSS) offers courses required for secondary school vocational–career–technical education teachers to become school-sponsored structured learning experience supervisors. The “Federal Wage and Hour and Child Labor Laws, Regulations and Hazardous Order Course” (FWH) was originally conducted in-person by U.S. Department of Labor-Wage and Hour Division from 2005 to Summer 2013, and then NJSS began conducting this course in-person (October 2013–April 2015). Staring in March 2015, this course was conducted online; beta-/pilot tests were conducted in Winter 2014–2015. Starting in May 2015, this course was offered exclusively online. This paper analyzes data from the in-person and online versions of the FWH, including overall course evaluation data comparing two versions with similar questions/constructs.

**Methods:**

The New Jersey Safe Schools Program modifications to FWH included adding information regarding the Fair Labor Standards Act’s Section 14(c) and supplemental case studies. The online version included information/resources provided during the in-person training plus assessments to supplement each module; the online version was split into modules to allow participants scheduling flexibility. Participants were given multiple possible attempts to achieve a minimum passing grade of 70%, excluding two ungraded activities (crossword puzzles simply completed). Descriptive statistics evaluated user satisfaction online compared to the in-person version of FWH and performance on aforementioned online assessments replacing in-person discussions/interactions.

**Results:**

Between October 2013 and April 2015, 160 participants completed the training in person; 156 had complete data. Between April and November 2015, 78 participants completed the training online; 74 participants had complete data. Other enrolled participants were in progress (not done as of 12/23/2015). Overall satisfaction was similarly high for in-person and online versions of FWH; over 95% of responding participants recommended this course to colleagues. Course evaluations for in-person participants indicated 83% felt the course objectives were completely met, whereas 95% of the responding online cohort felt course objectives were completely met. Further analyses examined performance of online assessments regarding number of attempts and scores achieved and performance on highlighted questions in certain module lessons.

**Conclusion:**

Data suggested the online format as a viable alternative to an in-person version of this training and provided NJSS and agency partners with ideas on how modifications/improvements can be made.

## Introduction

There are currently 507 secondary schools in the State of New Jersey (NJ) where high school students are able to participate in vocational-career-technical CTE programs across 16 different career clusters (1). Training for teachers in secondary schools includes safety and health (S&H), federal and state child labor, wage and hour laws, as well as hazardous occupations orders (HOs) in the United States (U.S.) Fair Labor Standards Act (FLSA) and is conducted by the NJ Safe Schools Program (NJSS) in collaboration with an alliance of federal and state agencies in the U.S. Region II (2). The U.S. Department of Labor (USDOL), for example, has provided, in collaboration with NJSS, various trainings to improve knowledge of child labor laws, wage and hour laws, and S&H across various job professions. In recent years, however, due to sequestration related budget limitations (including cuts) and staff retirements, NJSS was asked to take over their portions of the training series. Since NJSS staff could not share anecdotal experiences from the field, various supplemental discussion-based activities with participating teachers were used instead.

Traditional classroom-based in-person training courses are administered without the predominant use of online technology. Information is given orally or in writing by a certified instructor, who sometimes may demonstrate where to find relevant information on agency websites, e.g., USDOL. The online training format differs as the content is delivered through the use of online platforms on multiple types of hardware – desktop and laptop computers, tablets, and smartphones – without any face-to-face interaction. For adult worker S&H, online learning (e-learning) has been conducted in the construction industry with an emphasis on fall prevention, equipment use, and work practices in Taiwan (3) and in the U.S. (4). Recently, online virtual, multi-dimensional (2-D to 3-D) environments have also been used with adult workers for specific S&H training purposes, e.g., use of machines and power tools to simulate in-person hands-on demonstrations and practice sessions (5).

Currently, a fast growing trend in education is online learning. Notably, online college enrollment has grown almost 30% since 2010 (6). Potential advantages to online learning – either in synchronous and asynchronous formats – may include: lower initial or capital costs and/or ongoing management costs; consistency in material taught; standardized delivery method(s); convenience for users/students, as information available is accessed anytime, anywhere (asynchronous format, with most information available anytime); and, the ability for students to dictate their pace of learning in consideration of course assignment deadlines (7, 8). Foundations funding educational initiatives to the public and non-profit sectors have also recognized the increasing use of online learning in primary and secondary education in the U.S. (9).

Recently, the United States Department of Education (USDOE) conducted an evaluation of 51 online learning studies, with results suggesting that students who took classes online performed better, on average, than those who took the classes in traditional classroom settings (10). Results were predicted based on the fact that the students in online classes had an increase in learning time, potentially more innovative curriculum, and the ability to collaborate and reflect on the lessons learned (10).

Across several different fields, current literature has suggested, in terms of performance, no statistically significant difference between online learning and classroom-based learning (11, 12), though some studies have noted how web-based instruction may lead to better results, e.g., higher test scores (13–16). Another paper discussed the initial development and implementation of a 10 module, four discussion board-based online graduate school level law course – open since 1999 – and plans to reorganize and modify it to address workload issues among both students and faculty (17). The focus of these previous studies, however, was performance in higher education, large corporations, and business settings, not primary or secondary education. To date, limited research on secondary school teacher education has only pertained to highly specific science, technology, engineering, and mathematics – commonly referred to as STEM – education subjects (8, 18–22) or to master’s level training of in-service secondary school teachers (23). Indeed, no journal papers have been published to date regarding online learning about child labor laws.

This paper’s first objective was to describe the creation/conversion process of a training originally conducted in person with agency staff [2005–2013 school years (2)] and then only with NJSS staff (October 2013–April 2015) to an online training platform led by NJSS staff (as of spring, 2015). It must be noted that new teachers who have not yet completed the NJDOE/NJSS structured learning experience (SLE) supervisor curriculum will also be able to take the online version of this particular training (2) in the future. The second specific objective of this paper was to compare data on participant’s overall satisfaction of the in-person version of this course to the online version of this course for the years it was managed and led by NJSS and summarize available participant activity/assessment data from first year (2015) of the online version of the course. Simultaneously, these available data allowed critical review of feedback generated from the first year of the online version of the course, to evaluate and improve upon components as necessary.

## Materials and Methods

### Course Format

New Jersey Safe Schools Program used an updated PowerPoint, based on a set of four approved USDOL PowerPoint files, new NJSS case studies created for the in-person training, and other components to create an online version of the in-person training. USDOL and other federal and state agency partners throughout the State of NJ and Region II of USDOL agreed with this plan consistently when discussed at quarterly meetings 2014–2015. The format was agreed to be similar to other NJSS online courses currently 2–3 h in duration (2). Specifically, the online course included a lesson plan and modular organization with the aforementioned new NJSS case studies, additional readings (majority from USDOL websites), activities – as “knowledge quizzes” and “crossword puzzles” – and an overall course evaluation. This online Federal SLE training was divided into 3 main parts with 10 total subsections. The 10 subsections were FLSA information, Section 14(c) regarding individuals with special health care needs, i.e., various disabilities impacting productivity; and, child labor laws and HOs for both agricultural occupations [HO(A)s, comprising one career cluster and eight program pathways] and for non-agricultural occupations (HOs relevant to the other 15 career clusters and various program pathways).

### Target Population

Next, the new online version of the “Federal Wage and Hour and Child Labor Laws, Regulations, and Hazardous Orders Course” was subjected to internal beta- and pilot testing by Rutgers University students in public health (three undergraduate students and then three graduate students, respectively) in April, 2015.

The final online version of the course was first completed by eight NJ teachers/supervisors representing seven different school districts (one participant did not identify representing school district). These teachers had initially signed up for the March, 2015 in-person version of the training, but, there was an inclement weather/schools closed day throughout NJ. These eight teachers were from school districts in Northern NJ (*n* = 5) and Central NJ (*n* = 2). They completed the course, coursework was reviewed, and course evaluations were examined.

Then, this online version of the course then became open to general SLE audience on May 4, 2015 and initial open enrollment continued through October 30, 2015. This current cohort was given through the last week of November 2015 to complete relevant coursework. Throughout this first open enrollment period, reminders were sent out in 1-month increments from time of registration. The total 2015 cohort included 78 participants who completed relevant coursework, and 74 had complete data for the present analyses.

It was of interest to explore the performance of these participants throughout the nine lessons, as well as to compare course evaluations of online participants and in-person participants who completed the “Federal Wage and Hour and Child Labor Laws, Regulations and Hazardous Orders Course” between October 2013 and April 2015, during which NJSS alone conducted these trainings.

### Data Management

Data were entered, managed, and coded into Microsoft Excel, and then in both Excel and SPSS v22. Descriptive statistics were first computed with a quiz score for each of the assessments included in this training (Table 1), and second for both the percent correct and number of attempts per assessment to achieve ≥70%; the latter was examined because some participants were able to move on a few times with >50% and/or made extra attempts to try and score 100%. It must be noted how a cut-off of ≥60% was also examined. This is because though teachers, supervisors, and administrators were taking this course, and professional credentialing exams typically use ≥ 70% as a pass/fail cut off, ≥60% is typically used in U.S. schools for students and adults. It should also be noted that the 3 regions of NJ, which comprises 21 counties, were defined as follows with 7 counties per region (listed alphabetically per region): Northern NJ (Bergen, Essex, Hudson, Morris, Passaic, Sussex, Warren), Central NJ (Hunterdon, Mercer, Middlesex, Monmouth, Ocean, Somerset, Union), and Southern NJ (Atlantic, Burlington, Camden, Cape May, Cumberland, Gloucester, Salem).

**Table 1:** **Lesson titles**.

Lesson	Title
1	United States (U.S.) Fair Labor Standards Act (FLSA)
2	Employment relationship and the determining factors under the U.S. FLSA
3	Internships, minimum wage, and pay deductions
4	Overtime pay, hours, and recordkeeping
5	Penalties, compliance assistance, enforcement, and tips for employers and young workers based on the U.S. FLSA
6	Overview of employment determinants of section 14(c) of the U.S. FLSA
7	Child labor requirements in non-agricultural occupations under the U.S. FLSA
8	The 17 non-agricultural Hazardous Occupation Orders (HOs)
9	Federal laws on child labor in agricultural occupations under the U.S. FLSA

## Results

Table 2 presents the average of the highest total score (percent correct) per lesson. The average of highest scores across lessons was 94.4% correct. The three lessons with the top average score were Lesson 5 (99.6%), Lesson 8 (99.2%), and Lesson 2 (98.4%). Lesson 1 (84.1%), Lesson 3 (89.0%), and Lesson 4 (92.1%) had the three lowest average total score per lesson. The average number of attempts per lesson did not exceed 3 attempts for any lesson; however, 1 participant had a maximum of 28 attempts in Lesson 9, suggesting possible online user difficulties. Lesson 9 demonstrated the highest average number of attempts of about 3 (2.7). Lessons 2 and 5 (1.4) had the lowest average numbers of attempts.

**Table 2:** **Average highest total score (percent correct) and number of attempts per lesson for participants**.

	Number of items in quiz	Average of highest score (% correct)	Maximum number of attempts	Average number of attempts
Lesson 1	10	84.1	7	2.0
Lesson 2	4	98.4	3	1.4
Lesson 3	10	89.0	5	2.2
Lesson 4	5	92.1	6	1.6
Lesson 5	1^a^	99.6	4	1.4
Lesson 6	3	96.8	6	1.6
Lesson 7	10	94.8	4	1.4
Lesson 8	10	99.2	11	1.6
Lesson 9	10	95.8	28	2.7
All lessons	63	94.4		1.8

*N = 74*.

*^a^One quiz item with four parts*.

Table 3 presents the average of the highest total score (percent correct) per lesson stratified by gender. The average of highest scores across lessons was 94.8% correct for females and 94.2% for males. The 3 lessons with the top average score for females were Lesson 2 (99.5%), Lesson 5 (99.5%), and Lesson 8 (99.1%), which were consistent with the top 3 lesson score averages of participants (*N* = 74 out of 78) aggregated. The consistent pattern of Lesson 2, Lesson 5, and Lesson 8 having the highest average score upon further stratification by gender suggests that the lesson content, module layout, and assessment type were more effective than other modules. The three lessons with the top average score for males were Lesson 8 (99.4%), Lesson 6 (96.9%), and Lesson 9 (96.0%), of which only one lesson (Lesson 8) was consistent with the top three lesson score averages of both females and males aggregated. However, the number of males (*n* = 20) compared to females (*n* = 54) is to be noted. Both females and males had relatively similar average attempts of 1.8 and 1.7, respectively, across the lessons of this training. Both females and males had the highest average number of attempts of 2.7 and 2.6, respectively, in Lesson 9, which suggested possible lesson difficulties or decreased attention while trying to finish the last lesson of the course. The lowest average number of attempts was observed in Lesson 2 (1.3) for females and in Lesson 7 (1.3) for males.

**Table 3:** **Average highest total score (percent correct) and number of attempts per lesson by gender**.

		Females (*N* **=** 54)			Males (*N* **=** 20)		
	Number of items in quiz	Average of highest score	Maximum number of attempts	Average number of attempts	Average of highest score	Maximum number of attempts	Average number of attempts
Lesson 1	10	84.4	7	2.0	86.5	4.	1.7
Lesson 2	4	99.5	3	1.3	95.0	2	1.5
Lesson 3	10	90.9	5	2.3	86.4	5	2.2
Lesson 4	5	91.9	6	1.5	92.7	3	1.6
Lesson 5	1^a^	99.5	4	1.4	100.0	2	1.4
Lesson 6	3	96.5	6	1.6	96.9	4	1.7
Lesson 7	10	95.2	4	1.4	94.4	2	1.3
Lesson 8	10	99.1	11	1.7	99.4	4	1.6
Lesson 9	10	95.9	28	2.7	96.0	7	2.6
All lessons	63	94.8		1.8	94.2		1.7

*^a^One quiz item with four parts*.

Table S1 in Supplementary Material presents the number and correct percent of question answers by the highest score among attempts and lesson number, stratified by the gender. In other words, as an expansion of Table 3, these data indicated the highest scoring question by lesson for both males and females. It should be noted how one participant was excluded from lesson 3 and two participants were excluded from lesson 7 for these analyses due to incomplete data.

It should be also noted how across gender stratums only one question out of the four lessons had an overall failing score (≤60%): question one from Lesson 4 with a score of 28.4%. The lowest overall question score for each individual lesson is as follows: 73.0% for question 9 in Lesson 1, 67.1% for question 7 in Lesson 3, 28.4% for question 1 in Lesson 4, and 69.4% for question 10 in Lesson 7.

Table 4 presents the average of the highest total score per lesson stratified by geographic region of NJ (Northern, Central, and Southern). The average of highest scores across the lessons was 94.4% for Northern NJ, 95.5% for Central NJ, and 90.9% for Southern NJ. Northern and Central NJ average scores across lessons were within 1% of each other while Southern NJ was approximately 4.0% lower. Note, however, the Southern NJ study subsample (*n* = 3) was small relative to both Central NJ (*n* = 28) and Northern NJ (*n* = 43). The lessons with the top average score for Northern NJ were Lesson 5 (100%), Lesson 8 (99.3%), and Lesson 2 (97.7%), which were consistent with the top three lesson score averages of females and aggregated participants. The lessons with the top average score for Central NJ were Lesson 2 (100.00%), Lesson 5 (99.1%), and Lesson 8 (99.0%), which were consistent with the top three lesson score averages of Northern NJ, females, and participants aggregated. It should be noted how the top scoring lessons within these regional stratifications follow the aforementioned trend noted within the aggregate and gender stratified results. The lessons with the top average score for Southern NJ were Lesson 5 (100%), Lesson 8 (100%), and Lesson 7 (98.3%), two of which (Lessons 5 and 8) were consistent with Northern NJ and Central NJ, females, and participants aggregated. The three NJ regions had similar average number of attempts between 1 and 3, across lessons: Northern NJ with average number of attempts of 1.6, Central NJ with average number of attempts of 2.0, and Southern NJ with average number of attempts of 2.0. Lesson 9 had the highest average of attempts per individual lesson for Central NJ and Southern NJ, while Lesson 3 had the highest average number of attempts per individual lesson for Northern NJ. Lesson 5 and Lesson 7 (each 1.4) had the lowest average number of attempts for Northern NJ, whereas Lesson 2 (1.3) and Lesson 5 (1.0) had the lowest average number of attempts for Central NJ and Southern NJ, respectively.

**Table 4:** **Average highest total score (percent correct) and number of attempts per lesson by NJ geographic region (Northern, Central, and Southern)**.^b^

		Northern NJ (*N* **=** 43)			Central NJ (*N* **=** 28)			Southern NJ (*N* **=** 3)		
	Number of items in quiz	Average of highest score	Maximum number of attempts	Average number of attempts	Average of highest score	Maximum number of attempts	Average number of attempts	Average of highest score	Maximum number of attempts	Average number of attempts
Lesson 1	10	84.6	7	2.0	86.8	4	1.9	73.3	3	1.7
Lesson 2	4	97.7	3	1.4	100.0	2	1.3	91.7	2	1.3
Lesson 3	10	89.4	5	2.1	90.5	5	2.5	82.5	4	3.5
Lesson 4	5	91.9	3	1.4	93.0	6	1.8	86.7	2	1.3
Lesson 5	1^a^	100.0	4	1.4	99.1	4	1.5	100.0	1	1.0
Lesson 6	3	97.5	4	1.5	95.8	6	1.9	88.9	4	2.5
Lesson 7	10	94.9	4	1.4	94.8	4	1.5	98.3	2	1.5
Lesson 8	10	99.3	4	1.4	99.0	11	2.0	100.0	2	1.7
Lesson 9	10	94.3	6	2.0	98.3	28	3.6	96.7	7	3.7
All lessons	63	94.4		1.6	95.2		2.0	90.9		2.0

*^a^One quiz item with four parts*.

*^b^Geographic regions defined by counties (Northern: Sussex, Passaic, Bergen, Warren, Morris, Essex, and Hudson; Central: Hunterdon, Somerset, Union, Middlesex, Mercer, and Monmouth; Southern: Burlington, Ocean, Camden, Gloucester, Atlantic, Salem, Cumberland, and Cape May)*.

*Minimum number of attempts made = 1*.

Table S1 in Supplementary Material presents the number and the correct percent of question answers by the highest score among attempts and lesson number, stratified by region of NJ. Thus, as an expansion of Table 4, these data indicated the highest scoring question by lesson for Northern, Central, and Southern NJ. It should be noted how one participant was excluded from lesson 3 and two participants were excluded from lesson 7 for these analyses due to incomplete data.

Overall, Southern NJ had the highest frequency of perfect question scores (100%) for each lesson as well as the highest frequency of failing question scores (≤60%). This may be attributed in part to the low study subsample (*n* = 3) from Southern NJ. Data from course evaluations (Table 5) from the online course (74 out of 78 participants) suggested that a majority of participants were either very satisfied (39.7%) or satisfied (55.1%) with the overall online course. A majority of participants agreed the course content was satisfactory – 41.0% of participants were very satisfied with the course content and 55.1% of participants were satisfied with course content. In addition, a majority of participants felt this course was easy to navigate, with over one-third (34.6%) being very satisfied with course navigability and over half (52.6%) being satisfied with course navigability. Only about 4% of responding participants were dissatisfied with the online course navigability. The only other content areas where any dissatisfaction was indicated were in course instruction (2.6% dissatisfied) and course organization (1.3% dissatisfied).

**Table 5:** **Online training participant (*n* = 78^a^) course evaluation summary, 3/2015–12/2015**.

	Course satisfaction *n* (%)^b^			
	Very satisfied	Satisfied	Neutral	Dissatisfied
Please indicate your level of satisfaction with the course content	32 (41.0)	43 (55.1)	3 (3.8)	0 (0.0)
Please indicate your level of satisfaction with the knowledge checks	28 (35.9)	43 (55.1)	7 (9.0)	0 (0.0)
Please indicate your level of satisfaction with the course activities	27 (34.6)	44 (56.4)	7 (9.0)	0 (0.0)
Please indicate your level of satisfaction with the additional readings provided	27 (34.6)	44 (56.4)	7 (9.0)	0 (0.0)
Please indicate your level of satisfaction with the course organization	39 (50.0)	35 (44.9)	3 (3.9)	1 (1.3)
Please indicate your level of satisfaction with the graphics and pictures	20 (25.6)	48 (61.5)	10 (12.8)	0 (0.0)
Please indicate your level of satisfaction with the course instructions	28 (35.9)	46 (59.0)	2 (2.6)	2 (2.6)
Please indicate your level of satisfaction with the course navigation	27 (34.6)	41 (52.6)	7 (9.0)	3 (3.8)
Please indicate your level of satisfaction with the online course overall	31 (39.7)	43 (55.1)	4 (5.1)	0 (0.0)

*^a^Course evaluations are anonymous; there is no way to extract which evaluation came from completed coursework, thus total (*N*) is 78, not 74*.

^b^No participants were “very dissatisfied.”

About half of the participants indicated that they were able to complete this online course in 4–5 h (48.7%). About one-quarter (24.6%) of participants indicated that they were able to complete it in less than 4 h, and the remaining participants (26.7%) indicated that it took them 6 h or more to complete.

Results from in-person evaluations revealed similar results for course satisfaction (Table 6). A majority of responding participants reported, across content areas, the in-person training as “excellent” or “good.” The highest ranking content area was “knowledge of topic by instructor” with an average score of 3.69 out of a 4-point scale; this is similar to a 5-point Likert Scale without a “neutral” option. The weakest content area for in-person training indicated by course evaluation was “usefulness of content in meeting your needs” – average score was 3.41.

**Table 6:** **In-person training participant course evaluation summary, 10/2013–4/2015**.

	Number of responses (*n*, of *N* **=** 156)^a^				Average score
	Poor (1)	Average (2)	Good (3)	Excellent (4)	
a. Preparation and organization of instructor(s)	–	9	48	99	3.58
b. Responsiveness of instructor(s) to questions and concerns	–	4	42	110	3.68
c. Knowledge of topic by instructor(s)	–	6	37	113	3.69
d. Communication and presentation skills of instructor(s)^b^	–	14	36	105	3.59
e. Usefulness of content in meeting your needs^b^	5	15	47	88	3.41
f. Quality of facilities	1	9	51	95	3.54
g. Overall value of workshop	1	13	51	91	3.49

*^a^These data represent in-person training dates that occurred on 10/16/2013, 12/4/2013, 3/6/2014, 4/9/2014, 4/30/2014, 5/22/2014, 6/26/2014, 7/30/2014, 12/3/2014, and 4/16/2015*.

*^b^For these questions, one person did not respond (*N* = 155)*.

Online participants (74 out of 78) were relatively more likely to recommend this course to others than in-person participants (156 out of 160), although both groups were highly satisfied with the course (Tables 5 and 6); about 95% or more of each group (97.2% of online participants, 94.7% of in-person participants) would recommend it to others. Online participants were relatively more likely to agree that course objectives were met, though both groups were highly satisfied (96.2% of online participants, 82.6% of in-person participants). For nearly each responding participant, these training objectives were partially to completely met (100% of online participants, 99.4% of in-person participants). Collectively, the results showed that the online format can be a viable alternative to the in-person format for this training on federal child labor and wage and hour laws.

## Discussion

Between October 2013 and April 2015, 160 participants completed this training in person, and 156 participants had complete data. Between April 2015 and November 2015, 78 participants completed it online, and 74 participants had complete data.

Overall, participants who took the online format of the course were more likely to recommend the course to others compared to those who completed the course in person. The online format participants were also either very satisfied or satisfied regarding course navigability and course content. This may be due to how taking this course online has provided teachers with the ability to go at their own pace; some teachers were able to move at a faster rate than the in-person course delivery allowed. Therefore, the online format provided teachers with the information in a potentially more time-effective manner, at a rate at which they could better absorb the presented information and resources. The online format also provided the opportunity to be completed over the course of a few days, preventing teachers from taking time off from work to complete this training. Thus, the online format also reduced logistical resource expenditures, such as travel time to and from in-person training.

However, limitations existed as this evaluation could only compare satisfaction between the two course formats based on similar overall course evaluation survey questions. Going forward, something to be further investigated is an assessment of knowledge and awareness gained in the online version of the course and the in-person format, if/whenever offered in person by NJSS again. This may also include the need to account for any university-based transitions between learning management systems for online learning. Nevertheless, results from this study suggested that the online format is a viable option to the in-person format of this training, as the online course captured the cornerstones of adult-learning principles, such as assignment completion and expert-led learning with peer learning implications in the form of case studies.

At present, this training – in person and online – has been worth 6.0 professional development units (PDUs) from the State of NJ *via* NJSS. In the future, based on the amount of time it took for the majority of participants to complete the online version of the course, it may be valid to discuss with NJDOE how this course as run by NJSS can be reduced to 5.0 PDUs.

## Conclusion

New teachers who will be tasked with supervising school-sponsored structure learning experiences across career clusters and program pathways and who have not yet completed the NJDOE/NJSS SLE supervisor curriculum will continue to take the online version of this specific NJ SLE training in the future, starting again in February, 2016. In the future, it may also be possible for teachers in other states to complete and benefit from the course. Data to date suggested that an online format can be a viable alternative to the in-person version of this training on federal laws concerning wages and hours worked, child labor, and hazardous occupations within agriculture and non-agricultural industries. This is because available course evaluation clearly suggested that participants in both formats were satisfied with integral components of the course, such as course content and course organization. In addition, the online course format also has produced more data, as written activities replaced in-person anecdotes/discussion, generating rich feedback for NJSS to continue to amend and improve upon the course. Moreover, this evaluation provided NJSS and agency partners with ideas – beyond incorporating changes to federal laws and newly available agency resources – on how course delivery modifications and content improvements can be made to this online training, and evidence for potentially changing the number of NJ approved PDUs awarded to teachers for this course from 6.0 to 5.0 PDUs.

## Ethics Statement

The New Jersey Safe Schools Program has Institutional Review Board (IRB) approval from Rutgers Biomedical and Health Sciences, New Brunswick/Piscataway, New Jersey (formerly UMDNJ) for training evaluation analyses and state law based injury surveillance data analyses (IRB approval # 021997W0383).

## Author Contributions

DS, AA, AP, LM, and SK contributed to the conception and design of this effort. DS, AP, and LM led data management and analyses, and the interpretation of results; AA assisted the manuscript. DS, AA, AP, LM, and SK contributed to drafting and revising the manuscript.

## Conflict of Interest Statement

The authors declare that the research was conducted in the absence of any commercial or financial relationships that could be construed as a potential conflict of interest.
