# Supplementary material for: Comparing Efficacy of Online and In-Person Versions of a Training on U.S. Federal Wage and Hour, Child Labor Laws, and Hazardous Occupations Orders for Secondary School Professionals
Source: Front Public Health. 2016 Apr 25;4:75. doi: 10.3389/fpubh.2016.00075 (PMC4842762; doi:10.3389/fpubh.2016.00075)
Supplement: Supplementary file 1 [file table_1.doc]

**Supplementary Material for:
Comparing Efficacy of Online and In-person Versions of a Training on U.S. Federal Wage and Hour, Child Labor Laws and Hazardous Occupations Orders for Secondary School Professionals**

Derek G. Shendell, D.Env, MPH^1,2,3*^ Alexsandra A. Apostolico, MPH^1^ Lindsey J. Milich,^1,4^ Alexa A. Patti,^1,2^ Sarah W. Kelly, MPH^1^

^1^ Rutgers School of Public Health (SPH), Center for School and Community-Based Research and Education (NJ Safe Schools Program), New Brunswick, NJ

^2^ Department of Environmental and Occupational Health, Rutgers SPH, Piscataway, NJ

^3^ Exposure Measurements and Assessment Division, Environmental and Occupational Health Sciences Institute, Rutgers Robert Wood Johnson Medical School, Piscataway, NJ

^4^ Department of Epidemiology, Rutgers SPH, Piscataway, NJ

**Supplemental Table S1:** Number and Percent Correct of Question Answers by the Highest Score among Attempts and Lesson Number, by Gender

|  | **Question Number** | **Total**  **N correct (%)** | **Male**  **N correct (%)** | **Female**  **N correct (%)** |
| --- | --- | --- | --- | --- |
| **Lesson 1**  **N=74**  **Female N=54**  **Male N=20** | 1 | 72 (97.3) | 19 (95.0) | 53 (98.2) |
|  | 2 | 73 (98.6) | 19 (95.0) | 54 (100.0) |
|  | 3 | 65 (87.8) | 16 (80.0) | 49 (90.7) |
|  | 4 | 67 (90.5) | 19 (95.0) | 48 (88.9) |
|  | 5 | 65 (87.8) | 16 (80.0) | 49 (90.7) |
|  | 6 | 46 (62.2) | 14 (70.0) | 32 (59.3) |
|  | 7 | 64 (86.5) | 19 (95.0) | 45 (83.3) |
|  | 8 | 57 (77.0) | 17 (85.0) | 40 (74.1) |
|  | 9 | 54 (73.0) | 15 (75.0) | 39 (72.2) |
|  | 10 | 58 (78.4) | 17 (85.0) | 41 (75.9) |
| **Lesson 3^*^**  **N=73**  **Female N=53**  **Male N=20** | 1 | 53 (72.6) | 14 (70.0) | 39 (73.6) |
|  | 2 | 69 (94.5) | 19 (95.0) | 50 (94.3) |
|  | 3 | 63 (86.3) | 16 (80.0) | 47 (88.7) |
|  | 4 | 72 (98.6) | 19 (95.0) | 53 (100.0) |
|  | 5 | 70 (95.9) | 19 (95.0) | 49 (92.4) |
|  | 6 | 55 (75.3) | 13 (65.0) | 42 (79.2) |
|  | 7 | 49 (67.1) | 11 (55.0) | 38 (71.7) |
|  | 8 | 68 (93.2) | 19 (95.0) | 48 (90.6) |
|  | 9 | 66 (90.4) | 18 (90.0) | 48 (90.6) |
|  | 10 | 69 (94.5) | 17 (85.0) | 51 (96.2) |

**Supplemental Table S1** (continued):

| **Lesson 4**  **N=74**  **Female N=54**  **Male N=20** | 1 | 21 (28.4) | 7 (35.0) | 14 (25.9) |
| --- | --- | --- | --- | --- |
|  | 2 | 74 (100.0) | 20 (100.0) | 54 (100.0) |
|  | 3 | 73 (98.6) | 20 (100.0) | 52 (96.3) |
|  | 4 | 73 (98.6) | 20 (100.0) | 53 (98.2) |
|  | 5 | 74 (100.0) | 20 (100.0) | 54 (100.0) |
| **Lesson 7^**^**  **N=72**  **Female N=53**  **Male N=19** | 1 | 72 (100.0) | 19 (100.0) | 53 (100.0) |
|  | 2 | 52 (72.2) | 13 (68.4) | 39 (73.6) |
|  | 3 | 70 (97.2) | 18 (94.7) | 52 (98.1) |
|  | 4 | 69 (95.8) | 17 (89.5) | 52 (98.1) |
|  | 5 | 71 (98.6) | 19 (100.0) | 52 (98.1) |
|  | 6 | 69 (95.8) | 18 (94.7) | 51 (96.2) |
|  | 7 | 64 (88.9) | 17 (89.5) | 47 (88.7) |
|  | 8 | 66 (91.7) | 16 (84.2) | 50 (94.3) |
|  | 9 | 67 (93.1) | 17 (89.5) | 50 (94.3) |
|  | 10 | 50 (69.4) | 12 (63.2) | 38 (71.7) |

*One individual excluded

**Two individuals excluded

**Supplemental Table S2: Number and Percent Correct of Question Answers by the Highest Score among Attempts and Lesson Number, by Region of New Jersey**

|  | **Question Number** | **Total**  **N correct (%)** | **Northern New Jersey**  **N correct (%)** | **Central New Jersey**  **N correct (%)** | **Southern New Jersey**  **N correct (%)** |
| --- | --- | --- | --- | --- | --- |
| **Lesson 1**  **N=74**  **North = 43**  **Central = 28**  **South = 3** | 1 | 72 (97.3) | 41 (95.4) | 28 (100.0) | 3 (100.0) |
|  | 2 | 73 (98.6) | 43 (100.0) | 27 (92.9) | 3 (100.0) |
|  | 3 | 65 (87.8) | 35 (81.4) | 26 (92.9) | 3 (100.0) |
|  | 4 | 67 (90.5) | 40 (93.0) | 25 (89.3) | 2 (66.7) |
|  | 5 | 65 (87.8) | 38 (88.4) | 26 (92.9) | 1 (33.3) |
|  | 6 | 46 (62.2) | 27 (62.8) | 18 (64.3) | 1 (33.3) |
|  | 7 | 64 (86.5) | 39 (90.7) | 22 (78.6) | 3 (100.0) |
|  | 8 | 57 (77.0) | 32 (74.4) | 24 (85.7) | 1 (33.3) |
|  | 9 | 54 (73.0) | 32 (74.4) | 20 (71.4) | 2 (66.7) |
|  | 10 | 58 (78.4) | 31 (72.1) | 24 (85.7) | 3 (100.0) |
| **Lesson 3^*^**  **N=73**  **North=43**  **Central=28**  **South=2** | 1 | 53 (72.6) | 31 (72.1) | 22 (78.6) | 0 (0.0) |
|  | 2 | 69 (94.5) | 39 (90.7) | 28 (100.0) | 2 (100.0) |
|  | 3 | 63 (86.3) | 36 (83.7) | 25 (89.3) | 2 (100.0) |
|  | 4 | 72 (98.6) | 42 (97.7) | 28 (100.0) | 2 (100.0) |
|  | 5 | 70 (95.9) | 41 (95.4) | 27 (96.4) | 2 (100.0) |
|  | 6 | 55 (75.3) | 31 (72.1) | 23 (82.1) | 1 (50.0) |
|  | 7 | 49 (67.1) | 29 (67.4) | 19 (67.9) | 1 (50.0) |
|  | 8 | 68 (93.2) | 42 (97.7) | 25 (89.3) | 1 (50.0) |
|  | 9 | 66 (90.4) | 40 (93.0) | 24 (85.7) | 2 (100.0) |
|  | 10 | 69 (94.5) | 40 (93.0) | 27 (96.4) | 2 (100.0) |

**Supplemental Table S2** (continued):

| **Lesson 4**  **N=74**  **North = 43**  **Central = 28**  **South = 3** | 1 | 21 (28.4) | 12 (27.9) | 9 (32.1) | 0 (0.0) |
| --- | --- | --- | --- | --- | --- |
|  | 2 | 74 (100.0) | 43 (100.0) | 28 (100.0) | 3 (100.0) |
|  | 3 | 73 (98.6) | 43 (100.0) | 27 (96.4) | 3 (100.0) |
|  | 4 | 73 (98.6) | 43 (100.0) | 27 (96.4) | 3 (100.0) |
|  | 5 | 74 (100.0) | 43 (100.0) | 28 (100.0) | 3 (100.0) |
| **Lesson 7^**^**  **N=72**  **North=42**  **Central=28**  **South N=2** | 1 | 72 (100.0) | 42 (100.0) | 28 (100.0) | 2 (100.0) |
|  | 2 | 52 (72.2) | 30 (71.4) | 20 (71.4) | 2 (100.0) |
|  | 3 | 70 (97.2) | 41 (97.6) | 27 (96.4) | 2 (100.0) |
|  | 4 | 69 (95.8) | 41 (97.6) | 26 (92.9) | 2 (100.0) |
|  | 5 | 71 (98.6) | 42 (100.0) | 27 (96.4) | 2 (100.0) |
|  | 6 | 69 (95.8) | 39 (92.9) | 28 (100.0) | 2 (100.0) |
|  | 7 | 64 (88.9) | 38 (90.5) | 24 (85.7) | 2 (100.0) |
|  | 8 | 66 (91.7) | 38 (90.5) | 26 (92.9) | 2 (100.0) |
|  | 9 | 67 (93.1) | 39 (92.9) | 26 (92.9) | 2 (100.0) |
|  | 10 | 50 (69.4) | 28 (66.7) | 21 (75.0) | 1 (50.0) |

*One individual excluded

**Two individuals exclude
